# Kinematics associated with foot pronation in individuals with patellofemoral pain syndrome: a case-control study

**DOI:** 10.1186/1757-1146-4-S1-O4

**Published:** 2011-05-20

**Authors:** Christian J Barton, Pazit Levinger, Kate E Webster, Hylton B Menz

**Affiliations:** 1 Musculoskeletal Research Centre, Faculty of Health Sciences, La Trobe University, Bundoora, Victoria 3086, Australia

## Background

Excessive foot pronation during gait is frequently linked to patellofemoral pain syndrome (PFPS) development, due a proposed coupling of increased foot pronation with increased tibial and femoral internal rotation. However, there is a paucity of research which has compared kinematics associated with foot pronation between individuals with PFPS and controls. The aim of this study was to compare forefoot, rearfoot and tibial kinematics associated with foot pronation between individuals with PFPS and controls.

## Methods

Twenty-six individuals with PFPS (5 males and 21 females) and 20 controls (4 males and 16 females) aged between 18 and 35 were recruited. Three dimensional motion data was collected during natural comfortable walking using the Vicon motion analysis system incorporating the Oxford Foot Model. Between-group comparisons were made for magnitude and timing of peak angles, and range of motion at the forefoot (dorsiflexion and abduction), rearfoot (eversion) and tibia (internal rotation).

## Results

The PFPS group exhibited a trend towards slower walking velocity, (p = 0.07) so due to the potential of this to influence kinematics, all comparisons between the groups were adjusted for velocity. The PFPS group demonstrated earlier peak rearfoot eversion relative to the laboratory (30.4% *versus* 35.3% of the gait cycle, p = 0.01) and relative to the tibia (32.7% *versus* 36.5% of the gait cycle, p = 0.03). Effect sizes for these timing differences were -0.83 (-1.42 to -0.21) and -0.66 (-1.24 to -0.05), respectively. No significant differences were found for any variables associated with forefoot or tibial motion.

## Conclusions

Earlier peak rearfoot eversion in individuals with PFPS may indicate more rapid foot pronation following heel strike when walking. Due to the potential influence of this on knee and patellofemoral joint loading, this may be a factor related to the pathomechanics of PFPS development.

